# Editorial: Altered Expression of Proteins in Cancer: Function and Potential Therapeutic Targets

**DOI:** 10.3389/fonc.2022.949139

**Published:** 2022-06-22

**Authors:** João Pessoa, Marta Martins, Sandra Casimiro, Carlos Pérez-Plasencia, Varda Shoshan-Barmatz

**Affiliations:** ^1^ CNC – Center for Neuroscience and Cell Biology, CIBB – Center for Innovative Biomedicine and Biotechnology, University of Coimbra, Coimbra, Portugal; ^2^ Instituto de Medicina Molecular João Lobo Antunes, Faculdade de Medicina, Universidade de Lisboa, Lisbon, Portugal; ^3^ Laboratorio de Genómica, Unidad de Investigación Biomédica en Cáncer, Facultad de Estudios Superiores Iztacala, Universidad Nacional Autónoma de México, Instituto Nacional de Cancerología (INCAN), Mexico City, Mexico; ^4^ Department of Life Sciences and National Institute for Biotechnology in the Negev, Ben-Gurion University of the Negev, Beer Sheva, Israel

**Keywords:** cancer, protein expression levels, up-regulation, down-regulation, therapeutic target

## Introduction

The design of innovative cancer treatments requires extensive characterization of the molecular and cellular alterations associated with tumor development and progression. Cancer cells show extensive alterations in protein expression levels, which are drivers of their malignant transformation. Proteins with altered expression levels in cancer are involved in protein synthesis and degradation, signaling and metabolic pathways, DNA repair, apoptosis, and other cellular processes, whose alterations cause tumor development and progression. Characterizing the mechanisms that lead to alterations in protein levels and their cellular effects is an invaluable tool for repurposing those proteins as drug targets. Examples of up-regulated proteins in cancer include the epidermal growth factor receptor 2 (HER2) and the vascular endothelial growth factor (VEGF). HER2 is up-regulated in several cancer types, including breast (1), gastroesophageal (2), and non-small-cell lung cancers (3), making it an effective drug target (4). VEGF is up-regulated in pancreatic (5), prostate (6), and colorectal cancers (7), among others. Its inhibition is also an effective anticancer treatment, through a decrease in tumor vascularization (8).These examples demonstrate the modulation of protein levels as an effective anticancer target, which is becoming widely used in patient treatments. These studies also encourage additional research to uncover and test novel up-/down-regulated proteins as potential new therapeutic targets.

The aim of this Research Topic is to provide an update on some of these cancer-altered expression proteins. It contains 39 articles that include 23 original studies, 14 reviews, and 2 mini-reviews. In the following sections, we provide an overview of the main outcomes of these studies, which pertain to a large variety of proteins and cancer types. They were grouped based on the physiological or pathological role of the protein under study in order to highlight its impact on cellular homeostasis and malignant transformation into cancer phenotypes.

## Messenger RNA Transcription, Processing, and Stability

The cellular levels of a specific protein are in part determined by the levels of its messenger RNA (mRNA). mRNA levels are determined by the proteins and RNAs that regulate their transcription, processing, stabilization, and translation. The expression levels of these regulatory proteins and RNAs are frequently altered in cancer, and these alterations contribute to abnormal expression levels of other proteins, which bring deleterious consequences to the cellular homeostasis.

The levels of mRNA transcription are affected by chromatin exposure, which is dependent on histone modifications. Wang et al. reviewed the alterations of the histone demethylase KDM4B in several cancer types. This protein is generally up-regulated in cancer, resulting in altered levels of transcribed mRNA and subsequently translated protein. Several inhibitors of this histone demethylase are also discussed in a therapeutic framework. Liu et al. reviewed the cancer-related alterations in post-translation modifications in bromodomain-containing protein 4 (BRD4). This protein, which is usually up-regulated in cancer, binds to histones to prevent DNA damage and promote RNA transcription. This review also discusses the prospective therapeutic targeting of its post-translational modifications in several cancer types.

The transcription rates of mRNAs are also modulated by transcription factors and other regulatory proteins recruited to genomic transcription sites. Damerell et al. showed that the c-Myc transcription factor, commonly amplified/overexpressed in cancer, can directly activate transcription of the T-box transcription factor 3 in human mesenchymal stromal/stem cells, promoting their malignant transformation into a sarcoma. Gao et al. studied the role of up-regulated LIM domain only protein 1 (LMO1), a transcription co-regulator, in glioma. Its silencing could inhibit tumor growth *in vitro* and *in vivo*. Zhang et al. uncovered the role of the mediator complex subunit 19 in hepatocellular carcinoma. This subunit of the mediator transcriptional co-activator is up-regulated in this carcinoma, and its knockdown inhibited cellular proliferation, migration, and invasion. He et al. elucidated the joint effect of the SOX9 transcription factor, ANXA2P2 long non-coding RNA, and miR-361-3p microRNA in resistance against cisplatin, a first-line chemotherapeutic agent in cervical cancer. Up-regulating miR-361-3p or down-regulating ANXA2P2 could decrease resistance to cisplatin through the down-regulation of SOX9. Concerning non-coding RNAs, Zhou et al. reviewed the oncogenic role and mechanism of the up-regulated metastasis-associated lung adenocarcinoma transcript-1 (*MALAT1*, a long non-coding RNA), as a microRNA sponge in non-small cell lung cancer.

Alterations in the cellular levels of splicing factors have also been found in cancer. She et al. reviewed that the serine/arginine-rich splicing factor 6 (SRSF6) is up-regulated and has an oncogenic role in several cancer types. Its prospective therapeutic inhibition was discussed. Moreover, an original research article by Yang et al. showed that the splicing factor proline and glutamine-rich (SFPQ) is up-regulated in lung cancer mesenchymal stem cells, where it promotes proliferation, invasion, and drug resistance. Its knockdown could inhibit *in vitro* cell proliferation and *in vivo* metastasis.

Disruption of the communication between the nucleus and cytoplasm can also be a cause of cancer. Bindra and Mishra reviewed the roles of nucleoporins (subunits of the nuclear pore complex) in cancer and other diseases. These proteins are usually up-regulated in different cancer types, affecting the functioning of the nuclear pore complex and compromising cellular homeostasis.

In the cytoplasm, the amount of protein translated from an mRNA is also limited by its lifetime, which is determined by its stability. Therefore, proteins involved in mRNA processing and stabilization can also affect the levels of other proteins. Ma et al. reviewed the role of TAR-DNA-binding protein-43 (TDP-43) in several cancer types. This DNA/RNA-binding protein participates in DNA repair, microRNA processing, and long noncoding RNA-binding, as well as in mRNA splicing, transport, and stabilization. Depending on the cancer type, this protein can be either up- or down-regulated, playing oncogenic or tumor-suppressor roles, through alterations in RNA metabolism or DNA repair. Moreover, Li et al. reviewed the roles of the RNA-binding motif (RBM) protein family in several cancer types. These proteins can affect mRNA transport, processing, stability, and translation. Depending on the cancer type, they can be either up- or down-regulated. Their potential therapeutic exploitation was also discussed.

These studies show that several proteins and non-coding RNAs, involved in DNA and RNA metabolism, have their expression levels frequently altered in cancer. These alterations contribute to changes in the levels of translated proteins and induce malignant transformations. The above-mentioned protein examples regulating chromatin condensation and repair, as well as mRNA transcription and splicing are generally up-regulated in cancer cells. Proteins related to mRNA stability can be up- or down-regulated, depending on the cancer type. Interventions to restore their physiological levels, through the up- or down-regulation, emerge as a promising therapeutic approach.

## Protein Folding and Degradation

The cellular levels of proteins are also affected by the stabilization of their folded conformations and by their programmed degradation.

Molecular chaperones play critical roles in ensuring correct folding and consequent protein functionality. Albakova et al. reviewed the roles of heat-shock protein 70 (HSP70) and heat-shock protein 90 (HSP90) in several cancer types. The molecular and cellular consequences of the up-regulated cytoplasmic, mitochondrial and endoplasmic reticulum isoforms were highlighted.

Physiological protein levels also frequently depend on their regulated degradation through the ubiquitin-proteasome system. Proteasomal protein degradation requires its previous ubiquitination. Alterations in ubiquitination levels can alter protein levels, with oncogenic consequences. Zhou et al. reviewed the oncogenic role of ubiquitin-specific peptidase 7 (USP7), usually up-regulated in several cancer types. Xie et al. reviewed the dysfunction of the neural precursor cell expressed developmentally down-regulated 4-like (NEDD4L) E3 ubiquitin ligase, another regulator of protein ubiquitination, which is down-regulated in several cancer types and up-regulated in a few others.

These three reviews show that proteins regulating the folding and degradation of other proteins are also extensively deregulated in cancer. Specifically, the molecular chaperones HSP70 and HSP90 are generally up-regulated, whereas the ubiquitin-specific peptidase 7 and the NEDD4L E3 ubiquitin ligase, both regulators of proteasomal-mediated degradation, are up- or down-regulated, depending on the cancer type. The prospective therapeutic targeting of these proteins was also discussed.

## Signaling Pathways

The research into the molecular mechanisms that drive cellular transformations into cancer phenotypes often uncovers alterations in signaling pathways. These alterations are correlated with altered expression levels of critical proteins.

Manore et al. reviewed the role of interleukin-6, Janus kinases, and the signal transducer and activator of transcription 3 (IL-6/JAK/STAT3) signaling pathway in breast cancer metastasis. IL-6, its receptor, and other components of this signaling pathway are up-regulated in breast cancer and are related to poor prognosis. Inhibitors of multiple components of the IL-6 signaling pathway have been evaluated as potential drugs. Xu et al. reviewed the role of the same signaling pathway in hepatocellular carcinoma, where its up-regulation is also related to malignant effects, including proliferation, metastasis, and drug resistance, making this pathway an attractive drug target against hepatocellular carcinoma.

Tang et al. identified six genes related to interleukin 2 and the signal transducer and activator of transcription 5 (IL-2/STAT5) signaling pathway, which were up-regulated in acute myeloid leukemia patients. Increased levels of phosphorylated STAT5 were detected in patient peripheral blood cells. Entinostat, a prospective chemotherapeutic drug, was shown to decrease viability of cell lines with high levels of phosphorylated STAT5. Up-regulation of this pathway was proposed as a prognostic model for acute myeloid leukemia and as a potential therapeutic target.

Bose et al. have shown that up-regulation of mucin 1 in pancreatic ductal adenocarcinoma cells switches the role of transforming growth factor β (TGF-β) from tumor-suppressive to tumorigenic. Under high levels of mucin 1, TGF-β activates the c-Jun N-terminal kinase (JNK) signaling pathway, which increases the levels of the c-Myc transcription factor and cell viability. Knockdown of mucin 1 decreased the phosphorylation of JNK and activation of the pathway.

Feng et al. have shown that coatomer protein complex subunit beta 2 (COPB2), a component of the vesicles that mediate transport between the endoplasmic reticulum and the Golgi apparatus, was up-regulated in prostate cancer. Up-regulation promoted cell proliferation and invasion through an increase in the mitogen-activated protein kinase (MAPK)/TGF-β signaling pathway. Knockdown of COPB2 inhibited invasion in prostate cancer cells and tumor growth in mice.

Li et al. uncovered the mechanism through which hypoxia promotes the progression and metastasis of lung adenocarcinoma. They showed that hypoxia induces up-regulation of Notch4 in cell lines. This up-regulated Notch4 activates the extracellular signal-regulated kinase (ERK)/JNK/P38 MAPK signaling pathway, promoting cell proliferation and migration and inhibiting apoptosis. Silencing of Notch4 reduced signaling pathway activation.

Zhan et al. showed that the up-regulation of hZIP1, a zinc transporter down-regulated in clear cell renal cell carcinoma, has a tumor-suppressive effect against this cancer type. Up-regulated hZIP1 induced the down-regulation of hypoxia-inducible factor-1α (HIF-1α), which decreased the activation of the nuclear factor kappa B (NF-κB) signaling pathway. This decrease suppressed cancer cell proliferation *in vitro* and tumor growth in mice.

Pang et al. showed that ethyl ferulate (an anti-inflammatory, antioxidant, and neuroprotective compound) has a tumor-suppressive effect against esophageal squamous cell carcinoma. Treatment of cell lines with this compound inhibited the mammalian target of the rapamycin (mTOR) signaling pathway. Inhibition of this pathway was correlated with decreased cell growth and mouse tumor growth.

The up-regulation of signaling pathways has been generally related to tumorigenesis. Nevertheless, Guo et al. showed that epirubicin, a chemotherapeutic drug, could enhance the anticancer effects of radioactive iodine (^125^I) in hepatocellular carcinoma through the enhancement of phosphorylation of key proteins of the JAK/STAT1 signaling pathway. This study puts forward this pathway as a potential protector against hepatocellular carcinoma.

These studies show that cancer phenotypes are frequently associated with the up-regulation of signaling pathways. Interfering with an up-regulated signaling pathway, through the down-regulation of critical protein components, is an attractive therapeutic opportunity. Nevertheless, as demonstrated by the last above-mentioned study, a therapeutic approach may exploit a signaling pathway to exert its effects.

## Metabolism and Cell Homeostasis

The alterations in signaling pathways observed in cancer cells bring extensive metabolic alterations. These alterations impact the cell cycle, apoptosis and consequently, disrupting cellular homeostasis. The disruption of these processes is related to abnormal levels of specific proteins.

Mitochondria play a central role in cellular metabolism. Xie et al. reviewed the roles of proteins affecting mitochondrial fusion and fission in the framework of chemotherapy resistance. Up- or down-regulation of these proteins promotes or suppresses tumor growth, depending on the protein and cancer type, and can be exploited by the cancer cell to resist drug treatment. These proteins are attractive drug targets. Zhu et al. found that 70% of solute carrier protein family genes were differently expressed in lung adenocarcinoma tumors, in comparison to surrounding healthy tissue. Six of these genes were used to build a prognosis monogram for patient risk classification.

Zhang et al. showed that the mRNA and protein levels of pyridoxine 5′-phosphate oxidase (PNPO), an enzyme in the metabolism of vitamin B6, were increased in several cancer types and correlated with poor prognosis. PNPO was proposed as a potential novel biomarker for prognosis in several cancer types. Yang et al. identified ten differentially expressed genes in endometrial cancer, which were related to glycolysis. One of the corresponding up-regulated proteins, cyclin-dependent kinase 1 (CDK1), was chemically inhibited in cultured cells, resulting in decreased cell proliferation, invasion, and migration. CDK1 was proposed as a potential drug target in endometrial cancer.

Shizhe et al. combined single-cell RNA sequencing with multicolor immunofluorescence staining to map the alterations in liver zonation in hepatocellular carcinoma. Liver zonation reflects the organ’s microanatomical structure and is related to differential metabolic activity. Formimidoyl transferase cyclodeaminase, aminolevulinate dehydratase, and paraoxonase 1 are liver-specific proteins that contribute to its zonation. Their down-regulation in hepatocellular carcinoma was correlated to tumor differentiation. Zhang et al. applied a combined transcriptomics and proteomics approach to uncover fourteen deregulated genes in gastric cancer patient samples. These deregulated genes were related to metabolic pathways. One of the corresponding proteins, branched-chain amino acid transaminase 2 (BCAT2), which converts branched chain amino acids into their corresponding alpha-ketoglutarate, was down-regulated in tumors. Its decreased expression levels were related to poor prognosis. Its up-regulation in gastric cancer cell lines could suppress their proliferative capacity.

Metabolic alterations can induce changes in the cell cycle. Sheng et al. reviewed the role of cyclin-dependent kinase 5 regulatory subunit-associated protein 3 (CDK5RAP3) in several cancer types. This protein, whose many cellular roles include cell cycle regulation, apoptosis, and signaling transduction, can be up- or down-regulated, depending on the cancer type. Without a universal role in cancer, it can be a tumor-suppressor or promoter.

Zhang et al. showed that, in a subset of lung squamous cell carcinoma patients, the fibroblast growth factor 19 (FGF19) was co-amplified with cyclin D1, resulting in the up-regulation of both proteins. Their combined inhibition showed improved anticancer effectiveness, in comparison to individual inhibition. This study exemplifies synergistic co-amplification of neighboring genes as a cancer-promoting event. Wang et al. reviewed the roles of pleckstrin-2 (PLEK2), including cell spreading, inflammation, and erythropoiesis. PLEK2 is generally up-regulated in several cancer types. This review also discusses its roles in hematological cancers, tumorigenesis, and metastasis. Importantly, small molecular inhibitors of PLEK2 were not yet developed, in part due to the lack of a full-length structure of this protein. Zhao et al. uncovered that myeloid differentiation protein 2 (MD2) was up-regulated in glioma and related to poor prognosis. This protein acts as a co-receptor of toll-like receptor 4 (TLR4) to mediate innate immune response. Silencing of MD2 in cell lines decreased the expression levels of cytokines related to immune infiltration.

Other proteins have decreased levels in cancer. Ou et al. showed that the expression levels of the mRNA of family with sequence similarity 107 member A (FAM107A) are generally decreased in several cancer types. The corresponding protein, which binds to actin filaments to remodel the cytoskeleton, was knocked-down in renal and bladder cancer cell lines, resulting in increased proliferation, migration, and invasion. This protein emerges as a novel potential tumor-suppressor. One of the causes of cellular homeostasis disruption in cancer is the inhibition of apoptosis. Zhou reviewed our latest knowledge regarding the action mechanism of bortezomib and lenalidomide. These compounds are the two drug types approved for the treatment of glioma. They work through the activation of down-regulated caspase 8 to stimulate apoptosis.

The cancer-associated alterations in metabolism and cell homeostasis are deeply related to alterations in protein levels, which can be up- or down-regulated. Increased or decreased levels of specific proteins are helpful in predicting patient prognosis and/or as therapeutic targets through their inhibition or activation. The lack of clear trends concerning protein up-/down-regulation demonstrates the molecular complexity of the cellular processes that drive tumorigenesis.

## Metastasis

Metastasis, the transfer of cancer properties to previously unaffected regions of the organism, is a major cause of cancer mortality. In this process, proteins also play critical roles.

Wang et al. showed that the MiR-29-3p microRNA was down-regulated in papillary thyroid carcinoma, while COL1A1 and COL5A1 mRNAs, which code for two types of collagen, the major component of the extracellular matrix, were up-regulated. Overexpression of this microRNA could inhibit cell proliferation and metastasis *in vitro* through the down-regulation of COL1A1 and COL5A1. This study identified this microRNA as a potential new drug to combat cancer through the inhibition of excessive collagen deposition in the extracellular matrix. Wang et al. showed that exosomal CD44 could be a vehicle for transmission of lymph node metastatic capacity between gastric cancer cells. CD44 is located on the cell surface, where it mediates cell-to-cell interactions. In this study, it was found to be enriched in exosomes, mediating the spread of metastatic capacity to primary gastric cancer cells in a process that requires increased fatty acid oxidation. Exosomal CD44 could be a non-invasive marker for gastric cancer with lymph node metastasis and a potential drug target. Mei et al. developed a monogram to predict the effect of epidermal growth factor receptor 2 (HER2) levels in lymph node metastasis in early gastric cancer. Increased levels of this protein become oncogenic, and are found in about 20% of gastric cancer patients, enabling its utilization for prognosis.

These studies bring mechanistic insight into the induction of metastasis by protein up-regulation. These observations put forward those proteins as potential diagnosis tools and potential therapeutic targets.

## Concluding Remarks

**Figure 1:**
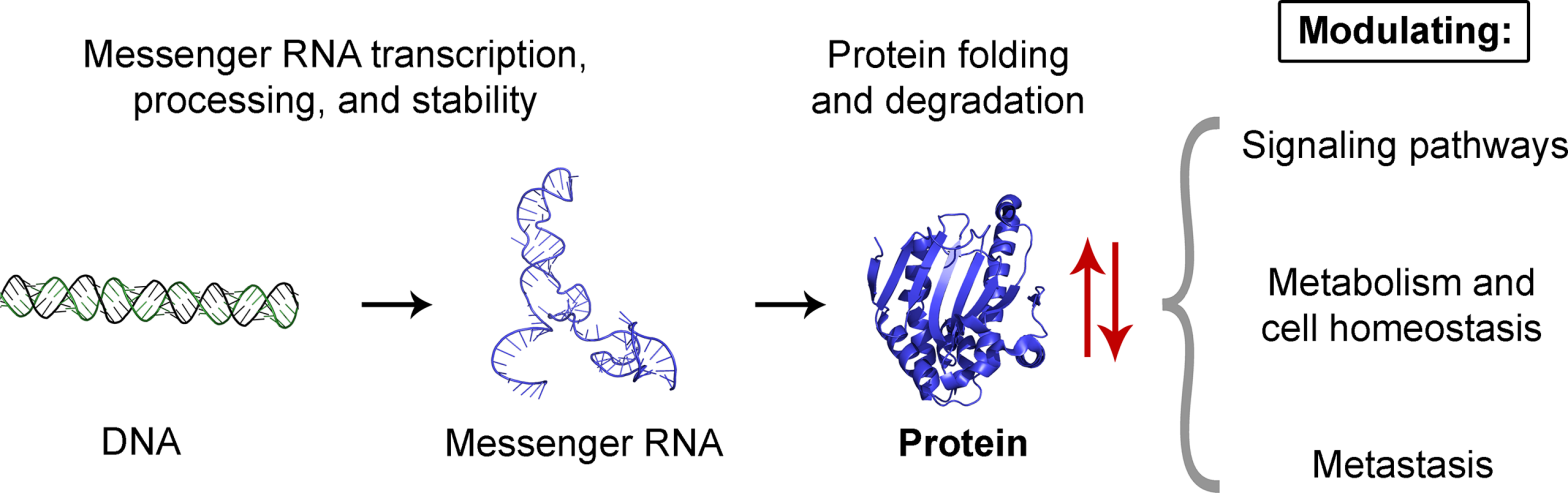
Impact of up-/down-regulated proteins in cancer. In cancer cells, alterations in protein levels (represented by red arrows) will be reflected in the cellular processes they control. These include mRNA transcription, processing, and stability, as well as folding and degradation of proteins that affect the cellular levels of other proteins. Moreover, alterations in protein levels affect signaling pathways and metabolism, which drastically change cellular homeostasis and promote metastasis. Elements are not drawn to scale. Protein and nucleic acid structure images are used for illustrative purposes only and were generated with PyMOL (Schrödinger, Inc.).

The 39 studies included in this Research Topic broaden our knowledge regarding the impact of up-/down-regulated proteins in cancer. These proteins, which are involved in mRNA transcription, signaling pathways, metabolism, and other cellular processes (**Figure 1**), provide opportunities for developing novel tools for diagnosis and therapy. These findings anticipate exciting achievements in the cancer field in the near future.

## Author Contributions

JP and VS-B wrote the article with input from MM, SC, and CP-P. All authors contributed insight and approved the final manuscript.

## Funding

This work was financed by the European Regional Development Fund (ERDF), through the COMPETE 2020 – Operational Programme for Competitiveness and Internationalization and Portuguese national funds *via* FCT – Fundação para a Ciência e a Tecnologia, under the projects POCI-01-0145-FEDER-028147 (VISCERAL), UIDB/04539/2020, UIDP/04539/2020, and LA/P/0058/2020 (to JP), and PTDC/MED-ONC/28636/2017 (to SC); by Programa de Financiamiento para la Investigación, UNAM, PAPIIT-IN231420, México (to CP-P); and by the National Institute for Biotechnology in the Negev (NIBN), Ben-Gurion University, Beer Sheva, Israel (to VS-B).

## Conflict of Interest

The authors declare that this work was conducted in the absence of any commercial or financial relationships that could be construed as a potential conflict of interest.

## Publisher’s Note

All claims expressed in this article are solely those of the authors and do not necessarily represent those of their affiliated organizations, or those of the publisher, the editors and the reviewers. Any product that may be evaluated in this article, or claim that may be made by its manufacturer, is not guaranteed or endorsed by the publisher.
